# Alliance of IVD manufacturer and medical laboratory for quality control of results

**DOI:** 10.1515/almed-2020-0079

**Published:** 2021-02-18

**Authors:** Marco Pradella

**Affiliations:** Italian Society of Clinical Pathology and Laboratory Medicine, SIPMeL, Castelfranco Veneto, Italy

**Keywords:** ISO 15189, quality control, ISO 15198

To the Editor,

The ISO 15189 standard in preparation [1] requires again monitoring of the performance of the methods, that is quality control (QC), as already defined by ISO 9000 in point 3.3.7 [2].

The Italian Society of Clinical Pathology and Laboratory Medicine (SIPMeL) Quality and Accreditations Commission produced its recommendations on this matter [3] acknowledging ISO 15198:2004 [4] document, confirmed for the first time in 2008 and revised with a positive result in December 2018. Key points are shared responsibility between the laboratory and the manufacturer, specific terms and definitions and validation and re-validation of updated quality-control procedures.

ISO 15198 is included in ISO package for medical laboratories, is taken up and re-launched in current ISO documents or even in preparation, in many documents of the Clinical Laboratory Standard Institute CLSI and in several documents of the World Health Organization (WHO) [3].

ISO 15198 reverses a commonplace widespread in medical laboratories. The responsibility for QC is shared between manufacturers and users of *in vitro* diagnostic medical devices. Identical principle is stated in ISO/TS 22367 (risk management) [5], [ 6] and in several points of ISO 15189. The so-called “individualized quality control plan” introduced in the USA as an alternative to the strict rules Clinical Laboratory Improvement Amendments has among the main objectives the strengthening of the collaboration between laboratory and manufacturer [7].

ISO 15198 incorporates terms already defined in several ISO and EN standards but adds new definitions: 3.5 control procedure, 3.7 examination procedure, 3.11 conditions of intermediate precision and 3.12 lot, batch.

QC in medical laboratories is often performed incorrectly. A survey on statistical quality-control practices in laboratories [8] describes the common use of the 1_2s_ control rule in most laboratories. In the survey “The Great Global QC Survey 2017” Westgard [9] observed that as many as 55% of laboratories use the 1_2s_ control rule for all tests. Half of the labs only examine the control once a day, before starting to process patient samples. Moreover, almost all use control materials prepared for them by the system manufacturers or by independent third parties, with or without assigned value. A Korean survey of small laboratories [10] showed that internal quality control is hindered by several factors, mainly by costs, lack of human resources or time, operational difficulties due to competence or informatic resources.

In ISO 15198 QC procedures point 4.1 and risk analysis point 4.2, the manufacturer must describe in the instructions for use the acceptable control materials, the frequency of examination of the control materials, the ways to establish the criteria for assessing the validity of the procedure of measurement and guidelines for actions to be taken on unacceptable quality-control results. Moreover, sufficient information to understand the fundamentals underlying the instructions must be provided.

QC procedures include a detection method (e.g. quality-control material, electronic monitoring system or internal chemical control) and acceptability criteria that will determine when a critical error occurs. The limitations of the QC procedure must be identified and described. The risk analysis method must consider the intended use of the device and the needs of the laboratory, identifies the sources of variability and potential hazards that are not mitigated by the device design or manufacturing process controls.

ISO 15198 requires validation of quality-control procedures (points 5.1–5.5), monitoring and revalidation (point 5.6). The validation protocol should include actual and/or simulated tests for error conditions. The manufacturer periodically assesses the adequacy of the recommended quality-control procedures, especially when changes are made to the design of the device, or adverse events are reported.

Enhancing the task of the diagnostic manufacturer has several advantages. It adds material and knowledge resources to those of medical laboratories, not always sufficient for these activities, which are often complex and expensive. It favors the harmonization of procedures between different laboratories and the comparability of the quality of the services provide, the emergence of specific critical issues, which reported at the manufacturer’s site and subjected to comparison with other laboratories, can find an adequate solution. SIPMeL therefore recommends that all diagnostic manufacturers provide the laboratory with appropriate internal quality-control instructions.
